# Nootkatone Is an Effective Repellent against *Aedes aegypti* and *Aedes albopictus*

**DOI:** 10.3390/insects12050386

**Published:** 2021-04-27

**Authors:** Taylor C. Clarkson, Ashley J. Janich, Irma Sanchez-Vargas, Erin D. Markle, Megan Gray, John R. Foster, William C. Black IV, Brian D. Foy, Ken E. Olson

**Affiliations:** Center for Vector-Borne Infectious Diseases, Department of Microbiology, Immunology, and Pathology, Colorado State University, Fort Collins, CO 80521, USA; taylorcclarkson@hotmail.com (T.C.C.); ajanich@rams.colostate.edu (A.J.J.); irma.sanchez-vargas@colostate.edu (I.S.-V.); erin.markle@ucdconnect.ie (E.D.M.); megnfreelance@hotmail.com (M.G.); foster.r.john@gmail.com (J.R.F.); william.black@colostate.edu (W.C.B.IV); Brian.Foy@ColoState.edu (B.D.F.)

**Keywords:** *Aedes* mosquitoes, nookatone, insecticide, repellent, Zika virus

## Abstract

**Simple Summary:**

Nootkatone, a natural organic compound in grapefruit and Alaskan yellow cedar, may have use as an insecticide and repellent against *Aedes* mosquito vectors of arboviruses. Here, we tested nootkatone against two medically important mosquito vectors, *Aedes aegypti* and *Aedes albopictus*. The insecticide potential of nootkatone was tested for both species using bottle bioassays and the repellency/irritancy and biting inhibition bioassays (RIBB) were used as tests for the *A. aegypti* strains only. We analyzed nootkatone’s insecticide potential against the New Orleans and Vergel strains of *A. aegypti* and ATM-NJ95 and Coatzacoalcos strains of *A. albopictus*. These strains were chosen because the New Orleans and ATM-NJ95 were permethrin susceptible (PERM-S) and Vergel was a confirmed permethrin resistant (PERM-R) strain. Coatzalcalcos was of unknown permethrin susceptibility. Permethrin is a commonly used insecticide to control mosquito populations, and permethrin resistance is becoming widespread in mosquito populations. We therefore wanted to compare nootkatone’s efficacy (and possible synergy) in the background of permethrin-susceptible and -resistant vectors. Bottle bioassays confirmed that the PERM-R Vergel strain was significantly less sensitive to nootkatone compared to PERM-S *A. aegypti* (New Orleans) and both *A. albopictus* strains were at least as sensitive to nootkatone as the New Orleans strain. We also showed that Zika virus (ZIKV)-infected New Orleans mosquitoes were as susceptible to nootkatone as the mock-infected controls. The infected Vergel strain was significantly less sensitive to nootkatone exposure than the New Orleans, ATM-NJ95, or Coatzacoalcos mosquitoes. In general, our studies concluded that as an insecticide, nootkatone was approximately 1000× less sensitive than permethrin, making it ineffective against *A. aegypti* and *A. albopictus.* However, RIBB analyses determined that nootkatone-treated arms of human subjects inhibited host-seeking and biting by *A. aegypti* mosquitoes. RIBB studies concluded that 20% nootkatone repelled mosquitoes at a rate comparable to commercially available *N,N*-Diethyl-*m*-toluamide (DEET; 7%) or picaridin (5%). Nootkatone has the potential to be an efficacious repellent against adult *Aedes* mosquitoes.

**Abstract:**

We tested a nootkatone product for insecticide activity against the most prominent vectors of Zika virus (ZIKV), *Aedes aegypti*, and *Aedes albopictus.* We tested the permethrin-resistant (PERM-R) Vergel strain of *A. aegypti* and the permethrin-susceptible (PERM-S) New Orleans strain of *A. aegypti* to determine if insecticide resistance affected their susceptibility to nootkatone. Bottle bioassays showed that the PERM-S strain (New Orleans) was more susceptible to nootkatone than the confirmed *A. aegypti* permethrin-resistant (PERM-R) strain, Vergel. The *A. albopictus* strain ATM-NJ95 was a known PERM-S strain and Coatzacoalcos permethrin susceptibility was unknown but proved to be similar to the ATM-NJ95 PERM-S phenotype. The *A. albopictus* strains (ATM-NJ95 and Coatzacoalcos) were as susceptible to nootkatone as the New Orleans strain. Bottle bioassays conducted with ZIKV-infected mosquitoes showed that the New Orleans (PERM-S) strain was as susceptible to nootkatone as the mock-infected controls, but the PERM-R strain was less susceptible to nootkatone than the mock-infected controls. Repellency/irritancy and biting inhibition bioassays (RIBB) of *A. aegypti* determined whether the nootkatone-treated arms of three human subjects prevented uninfected *A. aegypti* mosquitoes from being attracted to the test subjects and blood-feeding on them. The RIBB analyses data calculated the spatial activity index (SAI) and biting inhibition factor (BI) of *A. aegypti* at different nootkatone concentrations and then compared the SAI and BI of existing repellency products. We concluded that nootkatone repelled mosquitoes at a rate comparable to 7% DEET or 5% picaridin and has the potential to be an efficacious repellent against adult *A. aegypti* mosquitoes.

## 1. Introduction

*Aedes aegypti* and *Aedes albopictus* are mosquito vectors that transmit medically important arthropod-borne viruses (arboviruses), infecting millions of people across the globe. Both vector species transmit dengue, chikungunya, and most recently, Zika viruses (ZIKV) [1]. ZIKV caused a global pandemic in 2016 [2], leading to serious health consequences in infected humans. Arboviruses are transmitted to humans primarily through the bites of infected mosquitoes. No effective vaccines or antiviral therapies are available for widespread protection of humans from most arboviruses; thus, the primary method of disease control is to target the vectors with insect repellents and insecticides [3]. Alarmingly, *Aedes* mosquitoes are developing resistance worldwide to current insecticides and repellents due to their overuse [4,5,6,7]. There is a need for additional novel insect-control products that can be used as personal or household sprays, especially in areas where mosquitoes have developed resistance to insecticides and repellents [8].

Nootkatone, a sesquiterpene found in grapefruit and Alaskan yellow cedar [9] has been tested as a possible insecticide and/or repellent. Nootkatone is safely used in products such as juices and cosmetics to enhance flavor and fragrance [10], being that it is not known to be carcinogenic nor non-genotoxic, is approved for use in and on people, and is generally recognized as safe [11]. A naturally derived product, nootkatone may be more enticing to consumers than a synthesized compound like *N,N*-Diethyl-*m*-toluamide (DEET), an effective repellent sometimes associated with skin irritation, rashes, and swelling [12]. These side effects could potentially deter consumers with sensitive skin from using DEET. Nootkatone could offer consumers a favorable alternative to DEET-based products since nootkatone at >98% purity causes no skin sensitivity [13]. Nootkatone also shows potential as an effective acaricide for *Ixodes scapularis* ticks and insecticide for *Xenopsylla cheopis* fleas, *A. aegypti* adults [9], and *Coptotermes formosanus,* the Formosan subterranean termite [14].

For this study, we tested nootkatone’s efficacy as an insecticide against a permethrin-susceptible (PERM-S) and a permethrin-resistant (PERM-R) strain of *A. aegypti*. We also tested the insecticide potential of nootkatone targeting two *A. albopictus* strains. Repellency/irritancy biting inhibition Bioassays (RIBB) were used to test applications of nootkatone on human volunteers treated with different nootkatone concentrations prior to being exposed to PERM-S and PERM-R *A. aegypti* strains. Additionally, we compared nootkatone’s insecticidal capacity using ZIKV-infected and uninfected *A. aegypti* and *A. albopictus* to see if infected mosquitoes were killed as effectively as uninfected mosquitoes.

## 2. Materials and Methods

### 2.1. Mosquito Colonies

*Colony Maintenance:* Four mosquito colonies: *A. aegypti*, New Orleans (PERM-S), *A. aegypti*, Vergel (PERM-R), *A. albopictus*, ATM-NJ95 (PERM-S), and *A. albopictus,* Coatzacoalcos (unknown PERM sensitivity) were reared at the Arthropod-Borne Infectious Disease Laboratory at Colorado State University (CSU), Fort Collins, Colorado, USA. All mosquito colonies were maintained at a constant temperature of 28–29 °C and 70–80% relative humidity on an approximately 12 h:12 h light:dark cycle. Eggs were hatched in 150 mL of deionized room temperature water and larvae were fed fishfood daily. Pupae were placed in plastic cups within netted cages prior to their emergence as adults. Adult mosquitoes were given raisins *ad libitum.*

*Aedes aegypti*: New Orleans (PERM-S) mosquitoes were maintained in our insectary without exposure to insecticides. Vergel (PERM-R) mosquitoes of the 20th generation were used as a resistant colony [15]. Permethrin resistance was maintained in Vergel by exposing adults to permethrin every third generation. Permethrin concentrations ranged from 10 to 25 ug/bottle to select between 40% to 60% of the mosquitoes. The New Orleans (PERM-S) strain had no *kdr* alleles associated with knockdown resistance [6] and the Vergel (PERM-R) strain has been extensively characterized genetically and had an 88% frequency of the I1016 allele in the F_2_ and F_3_ generations [15,16,17].

*Aedes albopictus:* The ATM-NJ95 (PERM-S) population was obtained from BEI Resources (generation F_12_) as a confirmed insecticide susceptible PERM-S population of *A. albopictus* [18]. The permethrin susceptibility of *A. albopictus* collected from the city of Coatzacoalcos in Veracruz, Mexico was unknown but assumed to be more resistant than ATM-N95 due to the heavy use of pyrethroids in the collection region. Prior to performing the repellency or insecticide assays with nootkatone, all four colonies were tested in bottle bioassays to determine their current level of resistance (if any) to permethrin (see results). Coatzacoalcos proved not to have a significant resistant phenotype and the strain was similar to ATM-NJ95 in its sensitivity to permethrin (Supplementary Figures S1 and S2). *A. aegypti* and *A. albopictus* strains and their PERM-resistance status are summarized in Table 1.

### 2.2. Zika Virus Infection of Mosquitoes

Vero cells were infected with a Puerto Rican ZIKV (PRVABC-59-Asian) at a multiplicity of infection of 0.01, and the cells were incubated at 37 °C. ZIKV was harvested five days later from the culture supernatant to infect mosquitoes by intrathoracic injections. Adult mosquitoes were injected intrathoracically with 500 plaque-forming units (pfu) of ZIKV to ensure approximately 100% infection rates of all four *Aedes* strains. Injections of ZIKV were simpler and more efficient than infecting mosquitoes by the per os route. We inoculated 500 adult female mosquitoes (five days postemergence) using a Nanoject II (by Drummond Scientific Company, Broomall, PA, USA) to deliver 69 nL of PRVABC59 (500 pfu). A similar number of each mosquito strain was injected with only cell culture medium to serve as a control group. Mosquitoes were maintained in the insectary as previously described. At seven days postinfection, 30 infected mosquitoes from each group were analyzed for virus titer.

### 2.3. Bottle Bioassay: Mosquito Kill Assay

*Nootkatone concentrations:* Nootkatone powder (98%) was obtained from Evolva (Duggingerstrasse 23 CH-4153 Reinach Switzerland) for use in the bottle bioassays. Nootkatone powder was mixed with acetone solvent to apply nootkatone and fully coat the interior of 250 mL Wheaton bottles. A 1.0% nootkatone stock solution was prepared by dissolving 1.0 g of nootkatone powder in 99 mL of 100% acetone and used within one month of its preparation. The stock solution was assayed by mass spectrometry (GC-MS) at the central instrument facility at CSU for nootkatone and stored in a light-proof bottle at 4 °C until needed for bottle preparation. The nootkatone concentrations used in the bottle bioassays were 0.1%, 0.25%, 0.50%, and 1.0% in solution (See Supplementary Table S1). Bottle bioassay methodology has been previously described [19].

*Bottle assays:* We tested five replicates of the four *Aedes* mosquito strains for each nootkatone concentration. Uninfected female mosquitoes (3–4 days postemergence) were transferred from the cages to treated assay bottles (10 female mosquitoes/bottle). We recorded any “knocked-down” or immobile mosquitoes at the beginning of the experiment. During the experiment, we observed and recorded the number of mosquitoes knocked down at 10 min intervals, allowing mosquito exposure to various nootkatone concentrations for a total of 60 min. Knockdown behavior in mosquitoes was defined as the inability to right themselves, an inability to walk normally, a rapid wing vibration without capability of flight, or sporadic flight with an inability to land upright. After 60 min of exposure the mosquitoes were placed in netted cardboard recovery cups and given a 10% sugar–water solution. The mosquitoes were then held under insectary conditions (28–29 °C and 70–80% RH). After a 24-h recovery period, the total number of dead mosquitoes within each cup were counted. Any mosquitoes that displayed normal flight behavior and could right themselves were considered as recovered from the treatment.

The same procedure was conducted for the ZIKV-infected mosquitoes and the mock-infected mosquitoes, but these mosquitoes were around 12 days postemergence (five days old when infected; tested at seven days postinfection) to ensure the virus had fully disseminated prior to testing. Infected mosquitoes were maintained in a BSL3 environment, chilled at 4 °C for several minutes, then transferred to assay bottles.

*Bottle bioassay statistical analysis:* We calculated the average cumulative mortality at each nootkatone concentration of the five replicates. We used R 3.3.1 statistical software to calculate the lethal concentration needed to kill 50% of the mosquitoes (LC_50_) and 90% of the mosquitoes (LC_90_) and their associated confidence intervals (CI). A dose response curve was created (using Sigma Plot) for each concentration by plotting proportional mortalities calculated from R 3.3.1. We also calculated and plotted the time needed to knock down 50% of the mosquitoes (KT_50_) for each concentration, based on the number of knocked-down mosquitoes observed during the 60-min assay.

### 2.4. RIBB Assay

The RIBB apparatus was designed as described in Denham et al., 2015 [16] (See Supplementary Figure S3) with appropriate modifications [20,21]. Approximately 30 female *A. aegypti* mosquitoes 4–9 days postemergence were sorted into treatment groups at least 24 h prior to the initiation of the RIBB assay, and mosquitoes were deprived of sugar and water sources three hours prior to conducting each assay. *A. albopictus* were not tested with this assay because their host-seeking response in the RIBB apparatus was poor and thus inadequate for robust statistical analysis. Mosquitoes were released into the central chamber of the apparatus where they were allowed to acclimate for one hour. Three human volunteers were used to attract the mosquitoes within the RIBB apparatus to the side chambers and let them blood-feed on their arms. Each volunteer tested each repellent formulation in three independent replicates performed on different days. The volunteer avoided using lotions or other scented creams, oils, or toiletry products on the day of the experiment. During the time that the mosquitoes acclimated to the cage, the volunteer wore disposable gloves on each hand and had one forearm treated with a nootkatone formulation by the technician running the test, while the other forearm was left untreated, and arms were switched between replicate tests performed on different days. Three pumps of the application spray (mean = 0.4176 g of liquid, 95% CI (0.3985, 0.4368) analyzed by mass spectrometry) were used to treat each volunteer’s forearm, and the researcher running the test used a gloved hand to spread the repellents evenly over the treated forearm. The repellent dried on the arm for 1 h prior to testing. We tested nootkatone formulations provided by Evolva at 5%, 10%, and 20% nootkatone as well as a vehicle-only control formulation without nootkatone (0%). We also tested three commercially available repellents, 29% DEET (Repel Sportsman Formula), 7% DEET (OFF! Family Care Insect Repellent IV), and 5% picaridin (OFF! Family Care Insect Repellent II), to compare with the various nootkatone concentrations.

At the end of the 1 h drying period post-treatment, the volunteer inserted an arm into the sleeve of each side chamber and began to breathe through bifurcated tubing to deliver their breath into both side chambers. Timing began once the gates separating the side chambers from the central chamber were opened. Mosquitoes could move freely between the chambers for 10 min and feed on the arm of their choice (treated or untreated); volunteers kept their arms still so as not to disturb the mosquitoes trying to blood-feed. After 10 min the gates were lowered, and the volunteers’ arms were removed. The number of starting mosquitoes and those that moved into each chamber, as well as the number of blood-fed mosquitoes in each chamber recorded.

*RIBB Assay Statistical analysis:* We analyzed RIBB assay data using Prism 8.1.0 software. For both analyses, data from mosquito movement and blood-feeding in each side chamber were calculated for each volunteer per independent replicate (n = 3), and then grouped across data from each volunteer (n = 9) because effects by volunteer did not significantly differ. For spatial repellency, we determined the percentage of mosquitoes that left the middle chamber and moved to either of the side chambers. A weighted spatial activity index (SAI) was measured by counting the number of mosquitoes that moved away from the treatment (movement to the untreated chamber) relative to the total number of mosquitoes that moved from the middle chamber. This is equal to [(*N_c_ − N_t_*)/(*N_c_ + N_t_*)] × [(*N_c_ + N_t_*)]/*N*], where N is the total number of mosquitoes, *N_c_* is the total number of mosquitoes in the control chamber, and *N_t_* is the total number of mosquitoes in the treated chamber. SAI values for this statistic can range from 1 to −1, with 1 being the highest level of repellency, −1 being the highest level of attraction, and zero being no net response [22]. A Wilcoxon signed rank test was first used to compare the medians of each treatment to zero (no net response). Subsequently, mean SAIs were compared using ANOVA and Tukey’s multiple comparison posthoc test to determine pairwise differences between individual treatments. For the blood-feeding analysis, the mean proportions and 95% confidence intervals of mosquitoes in each side chamber that had blood-fed on each arm (untreated or treated) were calculated. The data were compared as pairwise replicates of blood-feeding proportions on the untreated vs. the treated arm per volunteer, per replicate (n = 9 for each repellent). We used a paired t-test presented as the mean of the difference in blood-feeding proportions between blood-feeding on the untreated arm compared to blood-feeding on the treated arm, as well as by the percent reduction in blood-feeding (1 − (mean treated proportion/mean untreated proportion)). For all analyses, *p* < 0.05 was considered statistically significant.

## 3. Results

### 3.1. Verification of Permethrin Resistance

Bottle bioassays were performed to verify that the PERM-S strains (*A. aegypti* New Orleans) remained highly susceptible, and the PERM-R strains (*A. aegypti* Vergel remained resistant. We determined that the *A. aegypti* strains did indeed differ in mortality, with the Vergel strain being 17.27 times more permethrin-resistant than the New Orleans strain (Supplementary Figure S1).

**Figure 1:**
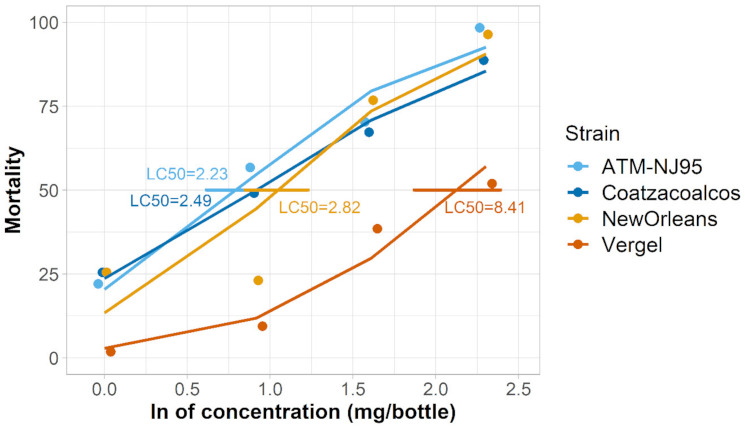
LC_50_ and 95% confidence intervals of four *Aedes* spp. when exposed to nootkatone. Points show the mortality caused by each concentration. Concentration–response lines were adjusted to a binomial logistic regression model. The LC_50_ for each strain is shown in mg/bottle.

**Figure 2:**
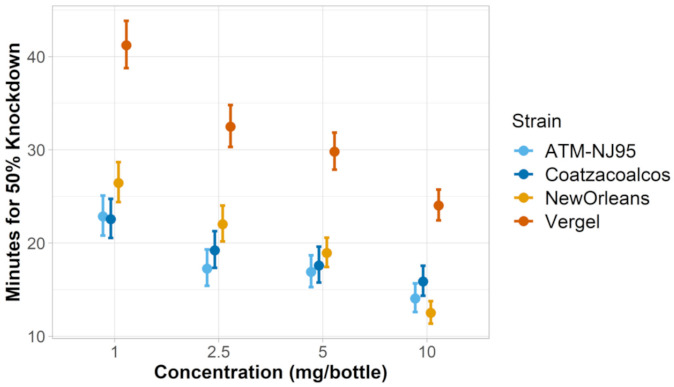
Knockdown time 50% (KT50) calculated for each concentration of nootkatone (mg/bottle) for *Aedes* spp. Whiskers denote the 95% confidence intervals. *A. aegypti* strains = New Orleans and Vergel and *A. albopictus* strains = ATM-NJ95 and Coatzacoalcos.

*Aedes aegypti strain sensitivity to nootkatone: A. aegypti* strains (New Orleans and Vergel) differed in nootkatone mortality as well as knockdown phenotype. When evaluating the LC_50_ of the two *A. aegypti* strains, we determined that approximately three times more nootkatone (resistance ratio [RR] = 3.11) was required to kill 50% of the Vergel mosquitoes than the New Orleans strain (Figure 1). When assessing knockdown, we determined that across all four concentrations of nootkatone, it required 1.47–1.92 times longer to reach 50% knockdown in the Vergel strain than the New Orleans strain, depending on the concentration (Figure 2).

*Aedes albopictus strain sensitivity to nootkatone:* The *A. albopictus* strains (ATM-NJ95 and Coatzacoalcos) had similar LC_50_s to nootkatone as the PERM-S New Orleans strain of *A. aegypti*. (ATM-NJ95: 2.50 mg/bottle, Coatzacoalcos: 2.49 mg/bottle, *A. aegypti* New Orleans: 2.23 mg/bottle). The confidence intervals (CIs) of the two *A. albopictus* strains were similar, but did not overlap with each other, suggesting a slight statistical difference (Table 2). By comparison, all three of these strains were significantly more susceptible to knockdown than the *A. aegypti* Vergel strain (Figure 1 and Figure 2 and Table 2).

**Figure 3:**
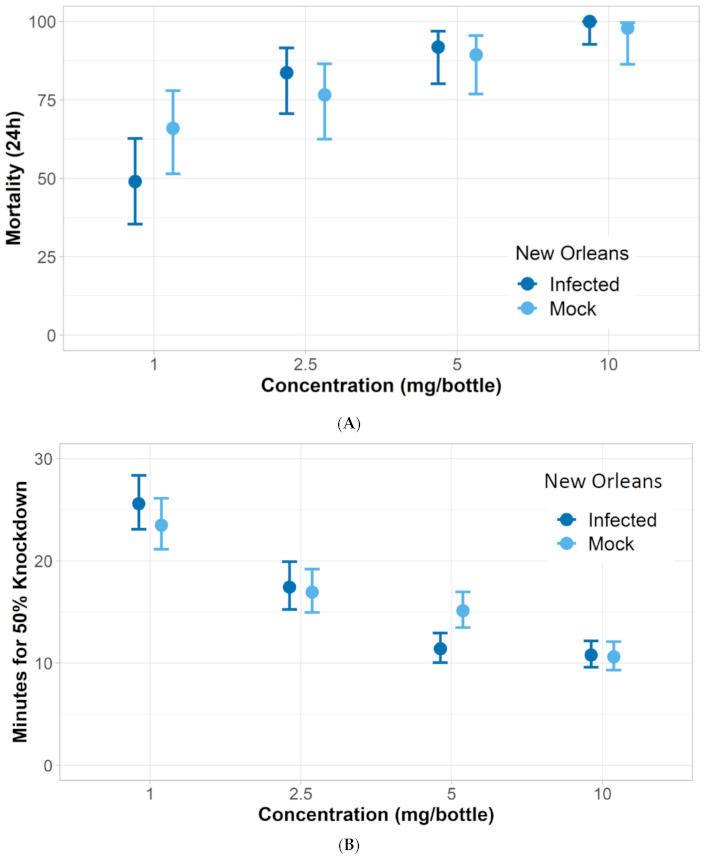
Response of New Orleans *A. aegypti* Zika-infected (infected) and uninfected (mock) mosquitoes when exposed to four concentrations of nootkatone (mg/bottle). (**A**) Mortality at 24 h and (**B**) 50% of knockdown time in minutes.

**Figure 4:**
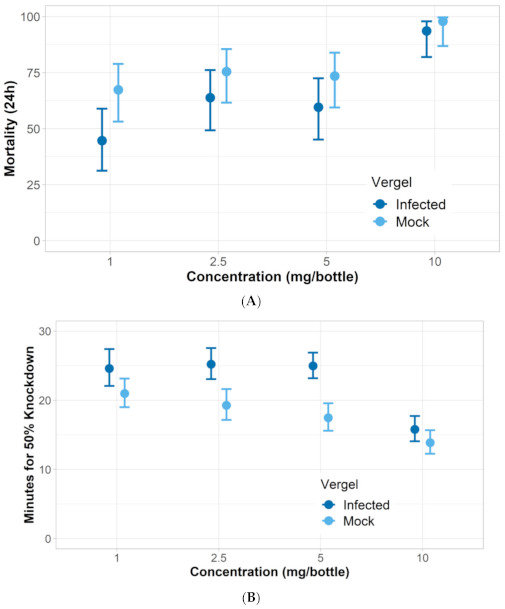
Response of Vergel *A. aegypti* Zika-infected (infected) and uninfected (mock) mosquitoes when exposed to four concentrations of nootkatone (mg/bottle). (**A**) Mortality at 24 h and, (**B**) 50% of knockdown time in minutes.

**Figure 5:**
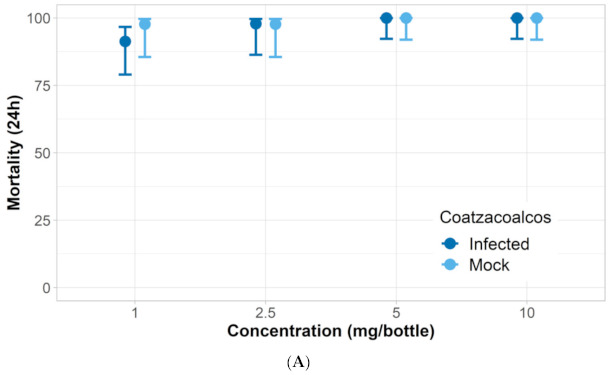

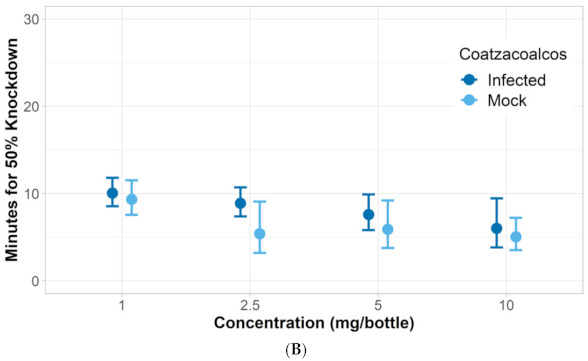
Response of Coatzacoalcos *A. albopictus* Zika-infected (infected) and uninfected (mock) mosquitoes when exposed to four concentrations of nootkatone (mg/bottle). (**A**) Mortality at 24 h and, (**B**) 50% of knockdown time in minutes.

**Figure 6:**
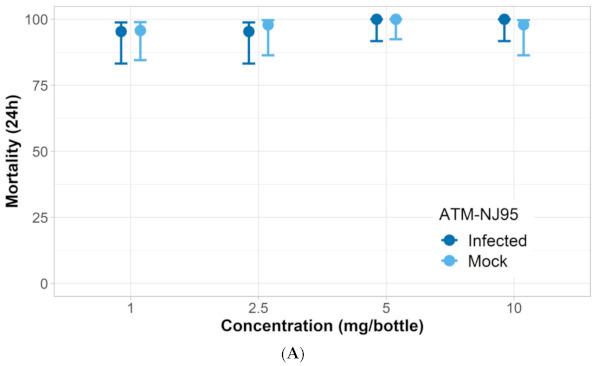

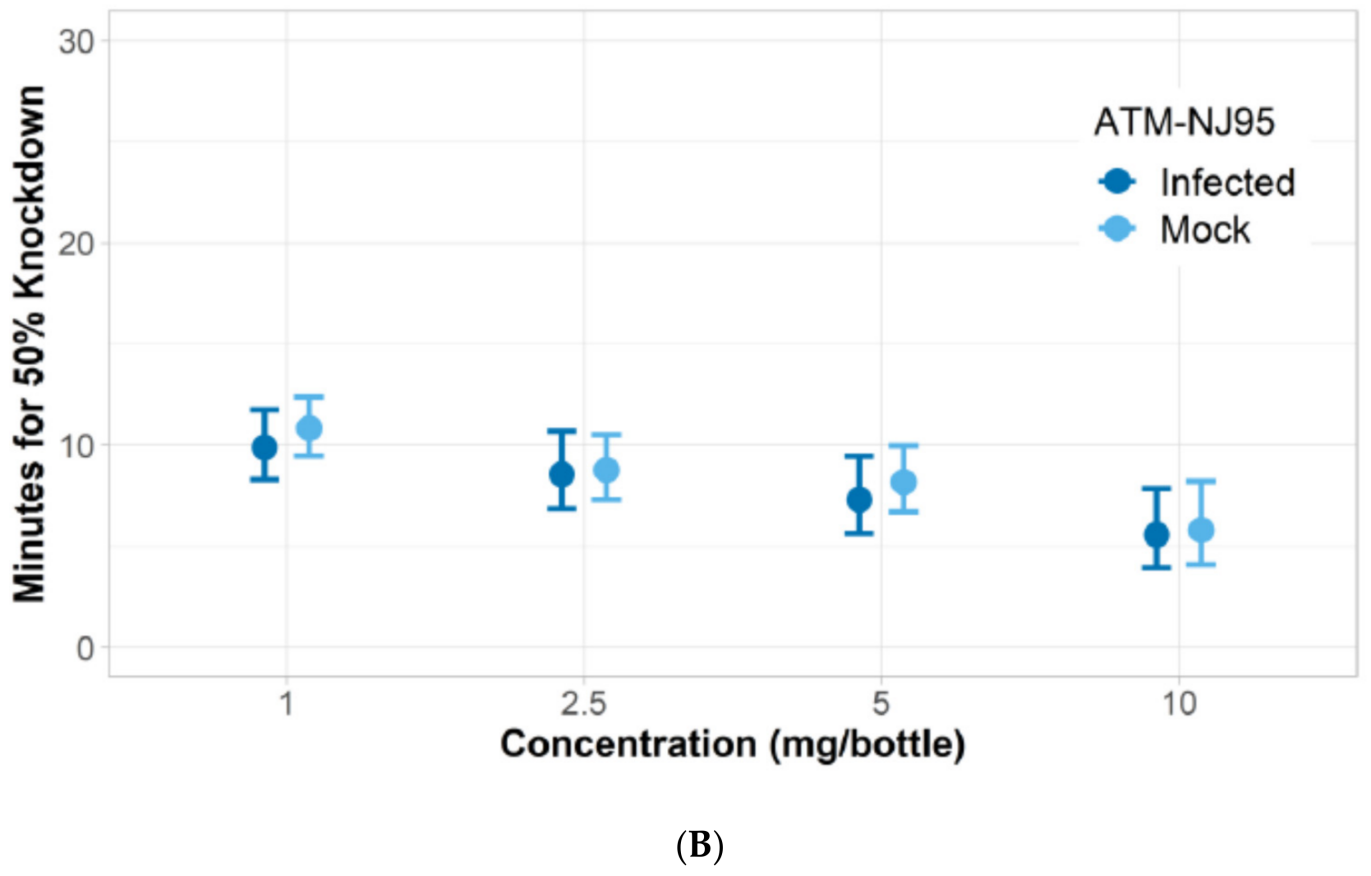
Response of *A. albopictus* ATM-NJ95 Zika-infected (infected) and uninfected (mock) mosquitoes when exposed to four concentrations of nootkatone (mg/bottle). (**A**) Mortality at 24 h and, (**B**) 50% of knockdown time in minutes.

*ZIKV-infected mosquito response to nootkatone*: Three of the ZIKV-infected *Aedes* strains had similar responses to nootkatone as mock-infected mosquitoes. The LC_50_ and KT_50_ from nootkatone exposure were statistically similar between mock-infected and ZIKV-infected groups for all strains except for Vergel, which had a higher LC_50_ and KT_50_ response for the ZIKV-infected group (Figure 3, Figure 4, Figure 5 and Figure 6). The ZIKV-infected Vergel strain also had the highest LC_50_ and KT_50_ compared to the other three ZIKV-infected strains (Figure 4 and Table 3). However, the LC_50_ of the mock-infected Vergel strain was similar to the mock-infected mosquitoes and the ZIKV-infected New Orleans strain, all of which had overlapping CIs (Table 3). The mock-infected New Orleans strain also had a comparable KT_50_ to the mock-infected Vergel strain (Figure 3 and Figure 4). While the KT_50_ of the ZIKV-infected Vergel was significantly higher than the mock-infected cohort (Figure 4 and Table 3), ZIKV-infected New Orleans and mock-infected *A. aegypti* responded similarly to nootkatone. The two *A. albopictus* groups, ATM-NJ95 and Coatzacoalcos, also responded similarly to each other, regardless of their infection status (Figure 5 and Figure 6 and Table 3). The two *A. albopictus* strains had lower KT_50_ values when compared to the *A. aegypti* strains (Figure 3, Figure 4, Figure 5 and Figure 6) and none of the *A. albopictus* had overlapping LC_50_ CIs with any *A. aegypti* strains (Table 3), indicating that the *A. albopictus* were more susceptible to nootkatone under these conditions.

### 3.2. RIBB Analysis of Nootkatone as a Personal Repellent for A. aegypti Mosquitoes

**Figure 7:**
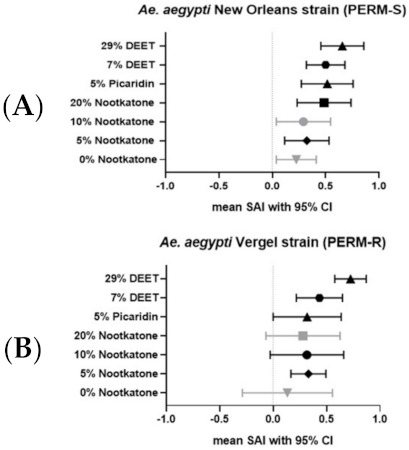
Mean spatial activity indices (SAI) for each of the seven treatment groups by strain of (**A**) *A. aegypti* New Orleans (PERM-S) and (**B**) *A. aegypti* Vergel (PERM-R) strains. SAI is expressed as the proportion of mosquitoes in the control relative to the treatment chamber after correcting for the proportion of mosquitoes in the control chamber. A positive value indicates repellency while a negative value indicates attraction and zero indicates no response. Whiskers designate the 95% CI. Gray plots indicate repellents formulations in which the SAI did not significantly differ from zero.

The spatial repellency assays gave positive spatial activity indices (SAI) for all tested formulations, indicating a slight bias towards repellency away from the treated arm. However, while SAI of the 0% (vehicle-only) nootkatone formulation was positive, this was not statistically significant. The spatial repellency effectiveness of nootkatone was mixed. Most nootkatone formulations significantly repelled both *A. aegypti* strains tested, but the SAI for 10% nootkatone formulation using the New Orleans (PERM-S) strain was not significantly different from zero, nor was the SAI for 20% nootkatone formulation using the Vergel (PERM-R) strain (Figure 7). All commercially purchased repellent formulations (5% picaridin, 7% DEET, and 29% DEET) significantly repelled both mosquito strains away from the treated arm. When compared, only 29% DEET significantly differed from 0% nootkatone using the New Orleans (PERM-S) strain, and from 0% and 20% nootkatone using the Vergel (PERM-R) strain (Tukey’s multiple comparisons test, *p* < 0.05).

Table 4 presents the effects of each mosquito repellent on mosquito blood-feeding. Regardless of strain, the two highest percentage nootkatone formulations along with the picaridin and two DEET formulations significantly inhibited mosquito blood-feeding when compared to the proportion that blood-fed on untreated arms in each replicate experiment, ranging from 24–88% inhibition. The 29% DEET was significantly more effective than all other formulations containing active ingredients at inhibiting blood-feeding on treated arms by the New Orleans (PERM-S) strain (0.11 vs. ≥0.56 mean proportions blood-feeding), but the inhibition was only significantly different from 5% nootkatone when using the Vergel (PERM-R) strain (0.18 vs. 0.65 mean proportions blood-feeding). When focusing on blood-feeding inhibition by the same repellent but between different mosquito strains, the only significant difference was between the proportions of New Orleans (PERM-S) strain blood-fed (0.94) compared to the proportions of the Vergel (PERM-R) strain blood-fed (0.65) on arms treated with 5% nootkatone.

## 4. Discussion

### 4.1. Nootkatone and Insecticide Bottle Bioassays

The bottle bioassays demonstrated that nootkatone treatment induced a dose-dependent insecticidal effect against the two *A. aegypti* strains compared to controls. However, the extent of knockdown and mortality due to nootkatone varied between the two *A. aegypti* strains. For example, the PERM-R Vergel strain required 8.34 mg/bottle of nootkatone to reach 50% mortality, nearly three times more than the PERM-S New Orleans strain. The mosquito strains’ responses to nootkatone followed a similar mortality pattern as permethrin, although Vergel was more permethrin-resistant than nootkatone-resistant. When analyzing knockdown, we saw that of the four concentrations of nootkatone tested, the Vergel strain required between 1.5 to 2 times longer to reach 50% knockdown compared to the New Orleans strain.

The *A. albopictus* strains had similar permethrin susceptibilities as well as nootkatone susceptibilities, which were also similar to the PERM-S *A. aegypti* New Orleans strain. This was surprising as we had originally assumed the Coatzacoalcos strain was PERM-R due to its collection from a region in Mexico that regularly uses permethrin and other pyrethroids for insect control. *A. aegypti* in this region have repeatedly shown pyrethroid resistance [23]. Other bottle bioassays that we completed in our laboratory showed that *A. albopictus* mosquitoes collected from Chiapas, Mexico (another region with high pyrethroid use) have low permethrin LC_50_ compared to *A. aegypti* collected from the same sites (data not published). It is possible that *A. albopictus* from these regions in Mexico respond differently to insecticides compared to *A. aegypti*, and further testing is needed to confirm this.

We observed a difference in response to nootkatone (KC_50_ and LC_50_) when testing with ZIKV-infected and mock-infected Vergel strain *A. aegypti*, but not with the ZIKV-infected and mock-infected New Orleans strain of *A. aegypti* or with ZIKV-infected and mock-infected *A. albopictus* (both strains). Other than the Vergel strain, the infection status of mosquitoes did not make a difference in their response to nootkatone. While infection with the virus may have impacted mosquito fitness, our observed differences with the infected Vergel strain suggest that this is due to its PERM-R status. Interestingly, mosquitoes that were simply injected with media or virus were more susceptible to nootkatone than those not receiving injections and had significantly lower LC_50_. We explain this in three ways. First, infected and mock-infected mosquitoes were a week older than those used in previously described assays due to the extrinsic incubation period required for virus replication and dissemination and older mosquitoes may be more sensitive to nootkatone. Second, we anesthetized infected and mock-infected mosquitoes at 4 °C prior to transferring them to bottles as a precautionary step to assure a safe work environment in the biosafety level 3 (BSL-3) laboratory. Third, injected mosquitoes trigger vector responses to wound healing that may alter the reaction to nootkatone.

With any insecticide, there is a risk of mosquitoes developing resistance from overuse. This may be especially true when considering the quantity of nootkatone that is required to achieve only 50% mortality. In our study, the New Orleans strain required nearly 1000 times greater concentration of nootkatone compared to permethrin in to achieve 50% mortality. Results from a previous study by Panella et al. [9], showed that nootkatone and several of its derivatives, along with several other essential oil extracts, could be potential pesticide candidates for vector control against *I. scapularis* ticks, *X. cheopis* fleas, and *A. aegypti* mosquitoes based on the comparative LC_50_/LC_90_ dose–mortality observations. However, there are significant differences between their mosquito bottle bioassay methodology compared to our standard, uninfected bioassays. Panella et al. used both male and female mosquitoes, older mosquitoes (5–7 days postemergence), more mosquitoes per bottle (25–50), and exposed the mosquitoes to the compounds for much longer (24 h), all of which could give lower LC_50_/LC_90_ values. These discrepancies in the insecticidal potential of nootkatone should be addressed in future studies with consistent methodology for bottle bioassay analyses.

Our data suggest that nootkatone efficacy was significantly reduced in PERM-R mosquitoes. Others have suggested that nootkatone has a different mode of action from pyrethroids because the sodium channel para-locus mutation in the *Anopheles gambiae* RSP-ST strain that confers permethrin resistance does not result in an increase in the nootkatone LC_50_ [24]. This group also concluded that nootkatone did not share a mechanism of action with organophosphates or carbamates [20]. However, in addition to target site mutations in the sodium channel gene that confer permethrin resistance, changes in detoxification proteins can also result in metabolic resistance to permethrin and other insecticides [25]. Perhaps the same enzymes that can break down permethrin can also break down nootkatone and may be why our results conflict with the McAllister and Adams study [24]. Another explanation for our conflicting results could be differences between the mosquito species tested (*Anopheles gambiae* vs. *Aedes aegypti*). Further studies are needed to investigate nootkatone’s target site and mechanisms of action in *Aedes* mosquitoes.

Based on our results that the Vergel strain showed less nootkatone susceptibility than the other strains, the potential for *Aedes* mosquitoes to develop resistance to nootkatone in regions already using pyrethroids for mosquito control may be strong. Future studies should investigate this potential and determine if nootkatone resistance can be artificially selected, as well as investigate whether cross-resistance to other insecticides may confer resistance to nootkatone. It would also be useful to know if there are synergists that are compatible with nootkatone and if those could improve nootkatone’s efficacy and reduce the amount needed to kill both insecticide-resistant and -susceptible mosquitoes.

### 4.2. Nootkatone and Repellency Assays

Our data broadly demonstrate that nootkatone exhibits spatial repellency and inhibits blood-feeding by *A. aegypti* mosquitoes. The spatial repellence effects we observed were modest, but not significantly different than the commercial picaridin or DEET products tested. Increased repellency trended along with increased concentrations of nootkatone, but only against the PERM-S strain. Similarly, blood-feeding inhibition was significantly different and most pronounced with the two highest percentage nootkatone formulations, comparing favorably with the lower concentration commercial picaridin (5%) and DEET (7%) formulations. However, the highest concentration of DEET (29%) was superior for inhibiting blood-feeding relative to all other repellent formulations tested when using the PERM-S strain, and nearly so when using the PERM-R strain.

While our data were well-replicated, and utilized three different human volunteers in the testing of each formulation, there still are some important biases. Among the three volunteers, there were no differences in mosquito movement or blood-feeding rates, but there was a marginal SAI difference from one volunteer relative to the other two (*p* = 0.0456). This can likely be attributed to different attraction/repellent factors existing among persons, such as their microbiome, scent, and diet [26]. While the RIBB assay is valuable for controlling for repellent effects on the same person with the same mosquitoes, it still assesses repellency and biting inhibition in a small, closed apparatus. Thus, our data should be compared against tests in larger cages or in field tests. There may also be inherent differences (other than permethrin susceptibility) relative to the strains that were used. The New Orleans strain is an established and well-adapted laboratory strain, while the Vergel strain was only taken from the field a few years prior to testing and so it is less adapted to the laboratory environment.

Finally, we did not test different time points post-application, and so we cannot provide evidence of the maintenance of nootkatone repellency or biting inhibition over time. A previous study found that natural repellents did not prevent landing or biting for longer than 30 min, while the highest protection and duration times were observed with DEET repellents [27]. They suggested that frequently reapplying natural-based products could help counteract the shorter duration times. A shortcoming often observed with plant-derived insecticides and repellents is that many of them are highly volatile and they may be lost as vapor over a short time [28,29]. Sesquiterpenes, like nootkatone, are generally less volatile than other plant-derived products like monoterpenes [29], but it remains unknown how long they can stay effective as repellents. It would be beneficial to investigate how long nootkatone can effectively repel mosquitoes and to determine how frequently the consumer would need to reapply it for continuous protection. During the production of this product, it would be beneficial to develop a way to increase the longevity of nootkatone. Possible ways to do this could be microencapsulation [30], combination with other, longer lasting chemicals, or to chemically modify nootkatone itself.

There is still a large knowledge gap regarding nootkatone. First, while nootkatone is approved as safe by the FDA, the amount used in food products is much lower than the amounts that would be required for use as a repellent or an insecticide. Additional research should be done to determine the impacts of high volume, long-term usage of nootkatone repellent spray on the environment and on humans, although it has been demonstrated that the chemical breaks down quickly in the environment and does not appear to negatively impact ecosystems [28,29]. In today’s market there is a growing trend towards “all natural” products [30] and an increasing demand for environmentally friendly insecticides and repellents [27]. People may have concerns about synthetic products, such as DEET. Nootkatone may be an appropriate substitute and potentially appeal to people who are reluctant to use, or who have shown skin reactions to such synthetic products.

## 5. Conclusions

Our findings suggest nootkatone is not an effective insecticide for *Aedes* mosquitoes in most applications due to the greater quantity of nootkatone required to achieve the same mortality rates as current insecticides, and the observation that permethrin-resistance in mosquitoes decreases their susceptibility to nootkatone. However, our data supports nootkatone use as an effective repellent. At a 20% concentration, nootkatone can significantly repel and reduce blood-feeding of *A. aegypti* mosquitoes at a rate comparable to other products such as 7% DEET and 5% picaridin, which are not marketed as all-natural products. We recommend marketing this product as an effective mosquito repellent, but not as an insecticide against *Aedes* mosquitoes.

## Figures and Tables

**Table 1:** Mosquito strains used to analyze permethrin resistance and reaction to Nootkatone.

Mosquito	Strain	Presumed	Actual	Reference
Species	-	PERM-status *	PERM-status **	-
*Aedes aegypti*	New Orleans	Susceptible	Susceptible	[5,6]
*Aedes aegypti*	Vergel	Resistant	Resistant	[15,16]
*Aedes albopictus*	ATM-NJ95	Susceptible	Susceptible	[18]
*Aedes albopictus*	Coatzacoalcos	Unknown	Susceptible	-

* PERM-status at the beginning of trials. ** PERM-status following bottle bioassays to analyze whether (or not) the mosquito colony was still resistant to permethrin.

**Table 2:** Summary of nootkatone LC50′s and corresponding 95% confidence intervals (CIs) among *Aedes* strains. Concentration–response was adjusted to a binomial logistic regression model (intercept and slope). Pearson goodness of fit (GOF), *p* value, and sample sizes are provided. Degrees of freedom = 1.

Species and Strain Name	LC50 (mg/Bottle)	95% CI (mg/Bottle)	Intercept	Slope	GOF	*p* Value	n
*A. albopictus* ATM-NJ95	2.24	1.85–2.71	−1.36	1.69	3.56	0.130	252
*A. albopictus* Coatzacoalcos	2.50	1.95–3.20	−1.17	1.28	0.48	0.850	217
*A. aegypti* New Orleans	2.83	2.34–3.42	−1.87	1.79	13.38	0.00	214
*A. aegypti* Vergel	8.42	6.49–10.91	−3.52	1.65	2.44	0.296	213

**Table 3:** Summary of nootkatone LC_50_s and corresponding confidence intervals (CI) among the four *Aedes* strains when ZIKV-infected or mock-infected.

Strain Name	LC_50_ (mg/Bottle)	95% Confidence Interval (mg/Bottle)
*A. aegypti* New Orleans infected	1.20	1.07–1.33
*A. aegypti* New Orleans mock	0.97	0.81–1.12
*A. aegypti* Vergel infected	1.87	1.68–2.06
*A. aegypti* Vergel mock	1.21	1.04–1.38
*A. albopictus* Coatzacoalcos infected	0.43	0.31–0.55
*A. albopictus* Coatzacoalcos mock	0.31	0.21–0.42
*A. albopictus* ATM-NJ95 infected	0.34	0.23–0.45
*A. albopictus* ATM-NJ95 mock	0.43	0.32–0.54

**Table 4:** Inhibition of *A. aegypti* blood-feeding in RIBB assay by tested repellents.

	*A. aegypti* New Orleans Strain (PERM-S)				*A. aegypti* Vergel Strain (PERM-R)			
Repellent Formulation	Mean Proportion BF on Untreated Arm (95% CI)	Mean Proportion BF on Treated Arm (95% CI)	Difference in Means (95% CI)	% Reduction	Mean Proportion BF on Untreated Arm (95% CI)	Mean Proportion BF on Treated Arm (95% CI)	Difference in Means (95% CI)	% Reduction
0% Nootkatone	0.90 (0.84, 0.97)	0.97 ^˄,↑^ (0.93, 1.02)	−0.03 (−0.09, −0.04)	−8%	0.92 (0.83, 1.00)	0.97 ^˄^ (0.93, 1.01)	−0.05 (−0.10,0.00)	−5%
5% Nootkatone	0.94 (0.88, 1.00)	0.94 *^,↑^ (0.86, 1.03)	0.00 (0.02, −0.03)	0%	0.90 (0.83, 0.96)	0.65 *^,˅↑^ (0.41, 0.89)	0.25 (0.42, 0.08)	27%
10% Nootkatone	0.88 (0.69, 1.07)	0.56 ^˅,↑^ (0.26, 0.87)	0.32 (0.43, 0.20)	36%	0.89 (0.77, 1.01)	0.42 ^˅^ (0.13, 0.70)	0.47 (0.63, 0.31)	53%
20% Nootkatone	0.95 (0.90, 1.00)	0.72 ^↑^ (0.53, 0.91)	0.23 (0.37, 0.09)	24%	0.95 (0.90, 1.00)	0.45 ^˅^ (0.22, 0.69)	0.50 (0.68, 0.32)	53%
5% Picaradin	0.91 (0.85, 0.97)	0.63 ^↑^ (0.41, 0.84)	0.28 (0.44, 0.13)	31%	0.81 (0.58, 1.04)	0.47 ^˅^ (0.19, 0.76)	0.34 (0.39, 0.28)	42%
7% DEET	0.94 (0.90, 0.98)	0.57 ^↑^ (0.27, 0.88)	0.37 (0.64, 0.10)	39%	0.92 (0.84, 1.01)	0.49 ^˅^ (0.23, 0.75)	0.43 (0.61, 0.25)	47%
29% DEET	0.95 (0.91, 0.98)	0.11 ^↓^ (−0.09, 0.32)	0.84 (1.01, 0.67)	88%	0.89 (0.78, 1.00)	0.18 ^˅,↓^ (−0.03, 0.38)	0.71 (0.81, 0.62)	80%

Proportions reported are the number of blood-fed (BF) mosquitoes in each side chamber of the RIBB assay after each 10-min test. Data are grouped from 9 replicate tests by 3 different human volunteers (n = 3 per volunteer). Bolded and italicized results indicate significant differences (*p* < 0.05) in means using pairwise t-tests. * indicate significant differences using unpaired t-tests in the mean proportions blood-feeding on arms treated with the same repellents between the two mosquito strains (in the same row). Opposite symbols (**^˄ ˅, ↑↓^**) indicate significant differences using Tukey’s multiple comparison tests following ANOVA in the mean proportions of the same strain mosquitoes blood-feeding on arms treated with different repellents (in the same column).

## Data Availability

The data presented in this study are available on request from the corresponding author. The data are not publicly available due to privacy concerns.

## Supplementary Materials

The following are available online at https://www.mdpi.com/article/10.3390/insects12050386/s1, Figure S1: LC_50_ and linear mortality regressions of Vergel and New Orleans *A. aegypti*. when exposed to permethrin were calculated in R 3.3.1. LC_50_s are denoted by vertical lines and are color coordinated with the respective strain, with LC_50_ values and CIs noted in the graph. Mortality is proportion of total mosquitoes that died at a given concentration of permethrin. Note that the scale is larger than the other graphs to accommodate for the large concentration needed to achieve nearly 100% mortality for the Vergel strain, Figure S2: LC_50_s and linear regressions of Coatzacoalcos and ATM-NJ95 *A. albopictus* when exposed to permethrin were calculated in R 3.3.1. LC_50_ are denoted by vertical lines and are color coordinated with the respective strain, with LC_50_ values and CIs noted in the graph. Mortality is proportion of total mosquitoes that died at a given concentration of permethrin. Our results indicated that Coatzacoalcos was not actually more resistant to permethrin compared to the susceptible control strain, ATM-NJ95, as seen by the overlapping Cis, Figure S3: RIBB apparatus. Tester arms go through the sleeves, the tester breathes through the bifurcated tube connected to both side chambers, and mosquitoes begin each experiment in the middle chamber, Table S1: Nootkatone (NK) bottle preparation. Amounts of 1% NK solution and 100% acetone needed to make the corresponding solution and bottle concentrations for the bottle assays.
