# Supplementary material for: Nootkatone Is an Effective Repellent against Aedes aegypti and Aedes albopictus
Source: Insects. 2021 Apr 27;12(5):386. doi: 10.3390/insects12050386 (PMC8145050; doi:10.3390/insects12050386)
Supplement: Supplementary file 1 [file insects-12-00386-s001.zip › insects-1059974-supplementary.pdf]

Table S1. Nootkatone (NK) bottle preparation. Amounts of 1% NK solution and 100% acetone needed to make the corresponding solution and bottle concentrations for the bottle assays.

NK Concentration (in Solution)	NK Concentration (in Bottle W/O Acetone)	Amount of 100% Acetone	Amount of 1% NK Solution
0%	0 mg	1000 μL	0 μL
0.10%	1 mg	900 μL	100 μL
0.25%	2.5 mg	750 μL	250 μL
0.5%	5 mg	500 μL	500 μL
1%	10 mg	0 μL	1000 μL

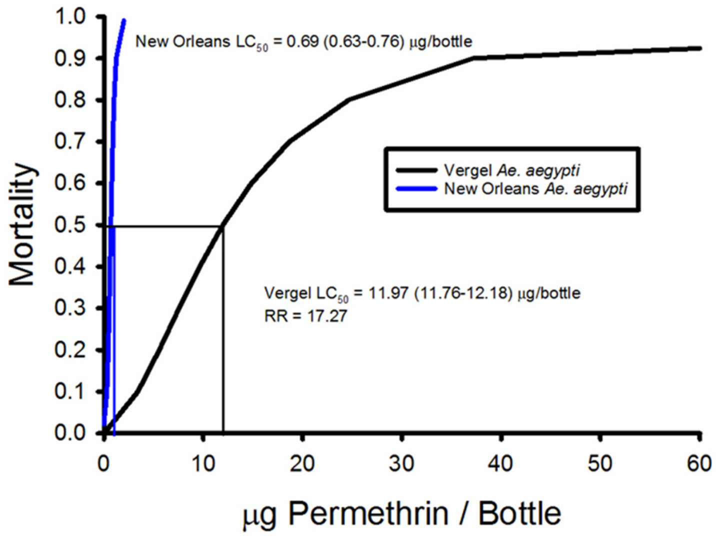

Figure S1. LC_{50} and linear mortality regressions of Vergel and New Orleans *A. aegypti*. when exposed to permethrin were calculated in R 3.3.1. LC_{50}s are denoted by vertical lines and are color coordinated with the respective strain, with LC_{50} values and CIs noted in the graph. Mortality is proportion of total mosquitoes that died at a given concentration of permethrin. Note that the scale is larger than the other graphs to accommodate for the large concentration needed to achieve nearly 100% mortality for the Vergel strain.

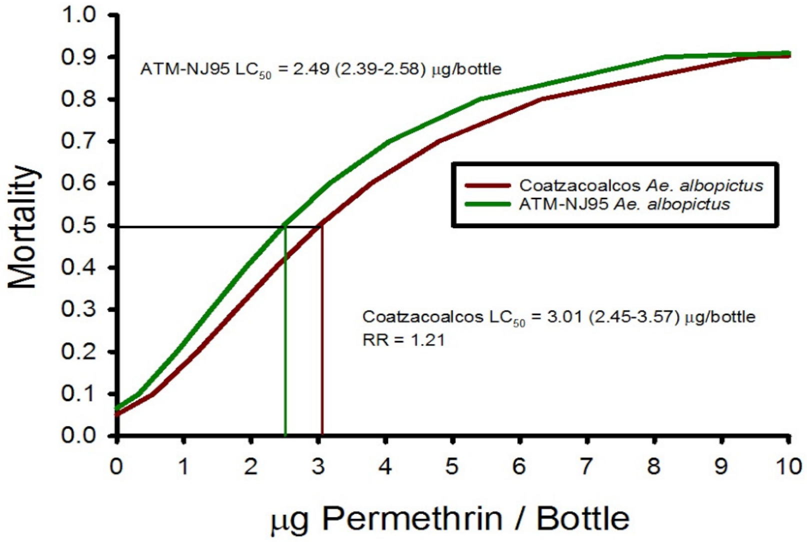

Figure S2. LC_{50}s and linear regressions of Coatzacoalcos and ATM-NJ95 *A. albopictus* when exposed to permethrin were calculated in R 3.3.1. LC_{50}s are denoted by vertical lines and are color coordinated with the respective strain, with LC_{50} values and CIs noted in the graph. Mortality is proportion of total mosquitoes that died at a given concentration of permethrin. Our results indicated that Coatzacoalcos was not actually more resistant to permethrin compared to the susceptible control strain, ATM-NJ95, as seen by the overlapping CIs.

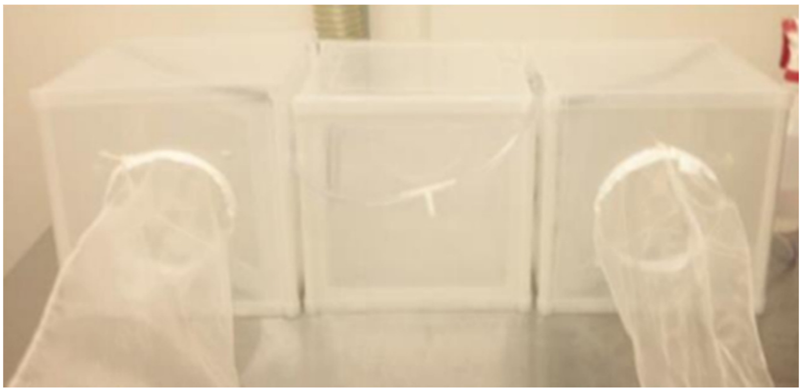

Figure S3. RIBB apparatus. Tester arms go through the sleeves, the tester breathes through the bifurcated tube connected to both side chambers, and mosquitoes begin each experiment in the middle chamber.
